# Taxonomic Guilds, Action Mechanisms, and Agronomic Deployment of Maize Rhizosphere Microbiota for Yield Enhancement: Unraveling G×M×E Interactions and Translational Barriers via Multi Omics

**DOI:** 10.3390/plants15213369

**Published:** 2026-11-01

**Authors:** Jie Lei, Na Lv, Ping Liu, Cheng Liu, Guoxiong Peng

**Affiliations:** 1 School of Life Sciences, Chongqing University, Chongqing 401331, China; 2 Chongqing Engineering Research Center for Fungal Insecticide, Chongqing 401331, China; 3 Chongqing Shapingba District Plant Protection and Inspection Station, Chongqing 400030, China; 4 College of Biological and Chemical Engineering, Chongqing University of Education, Chongqing 400065, China

**Keywords:** maize rhizosphere, plant growth-promoting microorganisms, multi-omics dissection, genotype–microbiome–environment interactions, microbial consortia

## Abstract

Maize (*Zea mays* L.) productivity is increasingly constrained by edaphic stressors—including soil salinization, drought, and acidification—posing mounting threats to global food security. Plant growth-promoting microorganisms (PGPM) offer a sustainable alternative to synthetic fertilizers, yet despite their pronounced performance under controlled laboratory settings, their beneficial effects often prove inconsistent in open-field environments, largely due to spatial heterogeneity and genotype-specific responses. Herein, we present a comprehensive synthesis of the core microbial guilds that have been robustly associated with maize yield enhancement. The mechanisms underlying these effects are examined across several dimensions, including biological nitrogen fixation, phosphate and potassium solubilization, phytohormone production, ethylene regulation, stress tolerance, and rhizosphere colonization. Genomic, transcriptomic, metagenomic, and metabolomic data are integrated to construct an interaction network between maize and rhizosphere microbes. We identify the genotype-by-microbiome-by-environment (G×M×E) interplay as the principal bottleneck hampering field-scale translational success. To address this issue, three priorities are proposed: (i) designing synthetic microbial consortia, (ii) using multi-omics to examine mechanisms, and (iii) developing tools for site-specific deployment. In conclusion, these sections provide a framework for developing more reliable microbial inoculants for maize.

## 1. Introduction

Maize (*Zea mays* L.) occupies approximately 200 million hectares of annual cropland worldwide, rendering it the most extensively cultivated cereal crop and one of the highest-yielding staple foods with the broadest consumption footprint [1]. Beyond its nutritional roles in human diets and animal feed, maize serves as a vital industrial feedstock; sustained demand, driven by both population growth and industrial expansion, further consolidates its strategic importance for global food security [1,2].

Nevertheless, maize cultivation is confronted with a multitude of abiotic constraints. The crop exhibits pronounced sensitivity to salinity, a concern magnified by the fact that nearly 20% of global arable land is currently affected by salinization—where osmotic imbalance, ionic toxicity, and oxidative bursts can collectively translate into yield penalties of up to 70% [3,4]. Drought represents another critical bottleneck. Ehinmitan et al. [5] projected that, under the worst-case scenario, just over half of the world’s cropland will experience severe water deficit by 2050. Meanwhile, the long-standing overreliance on synthetic fertilizers has progressively aggravated soil degradation, diminished nutrient-use efficiency, and exacerbated environmental pollution [6,7]. Compounding these issues, the scarcity of highly fertile lands has driven cultivation expansion toward acidic marginal soils, which are typically characterized by low pH, elevated concentrations of phytotoxic ions (e.g., Al^3+^ and Fe^2+^), and inherently poor nutrient availability [1,8].

The term plant growth-promoting microorganisms (PGPM) collectively designates a functionally diverse assemblage of beneficial microbes that colonize the rhizosphere and exert multifaceted effects on host plants—enhancing growth, facilitating mineral nutrient acquisition and utilization, and suppressing pathogenic organisms. This broad category encompasses two major taxonomic clusters: plant growth-promoting rhizobacteria (PGPR) and plant growth-promoting fungi (PGPF) [1,9]. The bacterial cohort, PGPR, comprises genera such as *Bacillus*, *Pseudomonas*, and *Azotobacter*, whereas its fungal counterpart, PGPF, encompasses arbuscular mycorrhizal fungi (AMF), *Trichoderma* spp., and other related taxa. The growth-promoting effects of PGPM on maize are mediated through a suite of complementary mechanisms, including biological nitrogen fixation, phosphate solubilization, secretion of phytohormones (e.g., indole-3-acetic acid and gibberellins), and reinforcement of host resilience against environmental stresses [3,10]. Moreover, integrated microbial-nutrient strategies are proving effective for disease management. For instance, the synergy between the biocontrol bacterium *Ochrobactrum ciceri* and zinc has been experimentally shown to suppress *Fusarium verticillioides* growth and mycotoxin production, while significantly improving maize seedling biomass under disease stress [11]. Functional strains isolated from stress-prone soils have demonstrated clear practical utility. For instance, drought-tolerant isolates of *Bacillus cereus* and *B. velezensis* have been shown to confer marked increases in germination rate, plant height, and above-ground dry biomass in maize [5]. In parallel, locally sourced *Bacillus paramycoides* and *Pseudomonas* strains, which are adapted to acidic conditions, exhibit a combinatory repertoire of nitrogen-fixing, phosphate-solubilizing, and IAA-producing traits; upon inoculation, these strains can reduce the requirement of synthetic fertilizer inputs by up to 50% [1,12].

**Figure 1:**
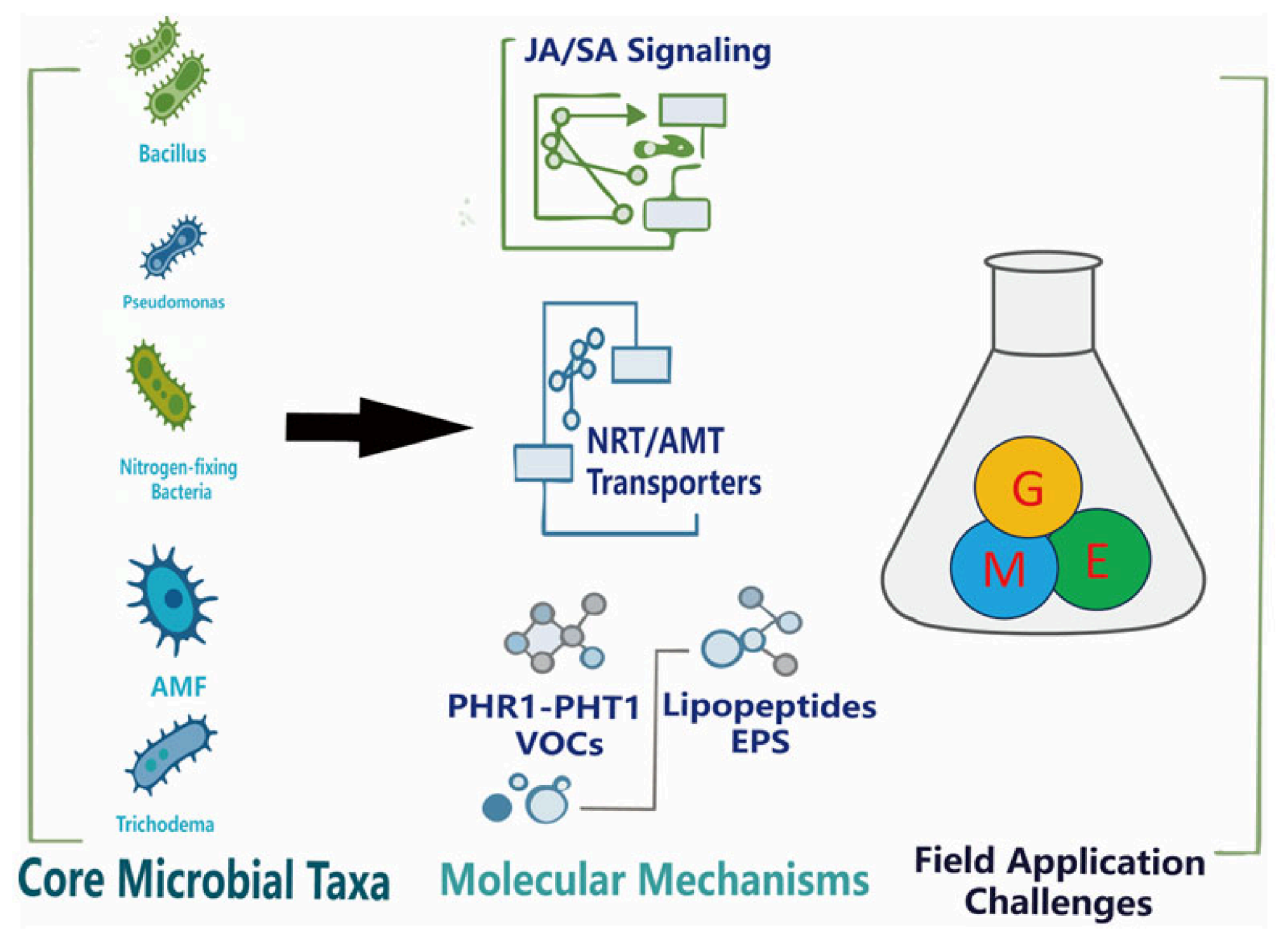
Integrative framework bridging rhizosphere microbial functional guilds, molecular signaling networks, and agronomic application bottlenecks. In this framework, established molecular mechanisms are represented in the central panel, proposed interactions are indicated by the directional flows, and field-level application challenges are spatially separated on the right panel.

While earlier reviews have thoroughly cataloged PGPM taxa and their isolated mechanisms, three critical knowledge gaps remain unaddressed: (i) the failure to distinguish gene presence from actual expression, biochemical activity, and demonstrated yield benefits; (ii) a lack of critical appraisal regarding the limitations of multi-omics integration (e.g., batch effects and correlation versus causality); (iii) the treatment of the genotype-by-microbiome-by-environment (G×M×E) interplay as a generic limitation rather than a testable framework. Central to addressing these gaps is the complexity of G×M×E interactions, which frequently undermines the field efficacy of otherwise robust laboratory strains due to competitive exclusion, niche overlap, and environmental fluctuations. To bridge these gaps, this review is structured along a four-tiered framework—functional guilds, molecular mechanisms, multi-omics networks, and field deployment—and integrates current multi-omics evidence to systematically dissect the action network and translational bottlenecks of maize rhizosphere growth-promoting microorganisms (Figure 1), with the overarching goal of informing the rational design and practical application of next-generation microbial fertilizers for maize.

### Review Methodology

This article is designed as a narrative review rather than a systematic review. To ensure a comprehensive and unbiased synthesis of the literature, we conducted a structured search across major databases, including Web of Science, Scopus, PubMed, and Google Scholar, covering publications from January 2015 to August 2026. The core keyword strings used for the search included: (“maize” OR “Zea mays”) AND (“plant growth-promoting” OR “PGPR” OR “PGPF” OR “rhizosphere microbiome”) AND (“multi-omics” OR “G×M×E” OR “field application”).

The inclusion criteria were peer-reviewed original research and reviews focusing on maize or closely related crops, specifically addressing rhizosphere microbial functions, molecular mechanisms, multi-omics, or field performance. Exclusion criteria included studies without clear microbial interventions, non-peer-reviewed preprints, and non-English publications. To assess evidence quality and avoid overgeneralization, we prioritized findings from high-impact journals, large-scale field trials, and multi-omics integration studies, while explicitly scoping conclusions derived solely from single-strain or controlled-condition experiments to reflect their specific boundaries.

## 2. Core Functional Guilds of Maize Rhizosphere Growth-Promoting Microorganisms

To date, over 30 genera spanning both bacterial and fungal domains have been documented as rhizosphere-dwelling microorganisms that possess plant growth-promoting capacities—with bacteria accounting for more than 20 genera and fungi for upward of 10 genera. In commercial agro-microbial formulations, *Bacillus*-based products constitute the predominant category, with *Pseudomonas* ranking second; among the fungal counterparts, *Trichoderma* and arbuscular mycorrhizal fungi (AMF) constitute the dominant groups. On a global scale, the AMF inoculant sector is expanding at an annual rate exceeding 15%, while the market for *Trichoderma*-derived products has surpassed USD 1 billion. These data indicate that *Bacillus* and *Pseudomonas* are the commercially predominant PGPR taxa, whereas AMF and *Trichoderma* are the most widely commercialized PGPF groups. Synthetic consortia are now widely used in agricultural practice [13,14].

### 2.1. PGPR

As highlighted above, plant growth-promoting rhizobacteria represent a core component of the rhizosphere microbial community, with *Bacillus*, *Pseudomonas*, and *Azotobacter* as the predominant genera. *Bacillus* spp. alone account for predominant category of global agro-microbial formulations, exerting their growth-promoting and biocontrol functions through the production of lipopeptide antibiotics, IAA, and siderophores [15]. Within this genus, *Bacillus velezensis* HN-Q-8 produces fengycin that achieves 70–90% inhibition rates against certain phytopathogenic fungi; moreover, targeted metabolic engineering—specifically, knockout of *srfAA* and *baeBE*—enhanced fengycin yield by 3.77-fold [16]. Strain 11B20, isolated from the maize rhizosphere, exhibits a triple-functional trait profile encompassing IAA production, phosphate solubilization, and siderophore secretion; in a greenhouse assay, inoculation with this strain resulted in a 48.2% increase in root length and a 35.4% increase in root dry weight [15]. Additionally, *Priestia megaterium* (formerly *Bacillus megaterium*) has been shown to suppress maize stalk rot and ear rot while concurrently reducing fumonisin B1 accumulation [17,18]. A 2026 investigation by Khuendee and colleagues demonstrated that *Priestia* sp. RMT2NF4 (a taxon recently reclassified from the *Bacillus* clade) markedly enhances early-stage traits, including seed quality and early-stage seedling vitality in sweet corn, while its full-genome analysis uncovered an intact repertoire of growth-promoting genetic determinants [19].

*Pseudomonas* spp. promote plant growth through siderophore-mediated iron acquisition, antibiotic-dependent pathogen suppression, and phytohormonal modulation [20,21]. As a representative, *P. putida* RB-2 exhibits a multifaceted growth-promoting trait profile encompassing ammonia production, phosphate solubilization, and IAA biosynthesis; when co-applied with organic fertilizers, this strain substantially enhanced chlorophyll content and nutrient uptake in maize compared to the application of organic fertilizer alone [22]. In the diazotrophic group, represented by *Azospirillum* and *Azotobacter*, growth promotion is achieved through biological nitrogen fixation combined with phytohormone production. Inoculation with *A. brasilense* Sp245 has been documented to increase crop yields by 5–15% while reducing nitrogen fertilizer requirements by 25%, accompanied by the upregulation of phosphate transporter genes *PHT1;1*, *PHT1;4*, and *PHO1* [23]. In *A. vinelandii*, more than 50 nitrogen fixation-related genes have been identified, and co-expression engineering has enabled a 10-fold enhancement in nitrogenase activity [24].

### 2.2. PGPF

The fungal cohort of growth-promoting microorganisms is predominantly represented by arbuscular mycorrhizal fungi (AMF) and *Trichoderma* spp. AMF establish symbiotic associations with over 80% of terrestrial plant species, extending the effective rooting zone through their extensive hyphal networks and thereby enhancing the acquisition of phosphorus, nitrogen, and other mineral nutrients [25]. Synergistic effects have been documented when AMF are co-inoculated with *Bacillus subtilis*: under conditions without external phosphorus fertilization, this dual inoculation boosted root and shoot dry weights by 3000% and 680%, respectively, relative to the control, while shoot phosphorus accumulation increased by 1700% [26]. Furthermore, the combined application of AMF and biochar has been shown to effectively alleviate salt-induced growth suppression in maize [27]. A 2026 study by Mohan and Joshi successfully obtained pure cultures of native AMF exhibiting high symbiotic compatibility with maize through single-spore propagation; inoculation with these isolates resulted in statistically significant increases in both shoot and root biomass in maize [28]. The genus *Trichoderma* fulfills a dual role in growth promotion and biological control. For instance, field application of *Trichoderma harzianum* K179 has been shown to reduce the severity of maize ear rot, enhance grain yield, and simultaneously decrease the accumulation of fumonisins and aflatoxins [29]. At the mechanistic level, the *T. harzianum*-derived cellulase Thph2 triggers a burst of reactive oxygen species via the jasmonic acid signaling pathway, leading to a 15.9% reduction in lesion area caused by southern corn leaf blight [30]. A summary of the core microbial taxa associated with maize yield enhancement and their underlying functional mechanisms is provided in Table 1.

## 3. Molecular Mechanisms Underlying PGPM-Mediated Yield Enhancement in Maize

**Figure 2:**
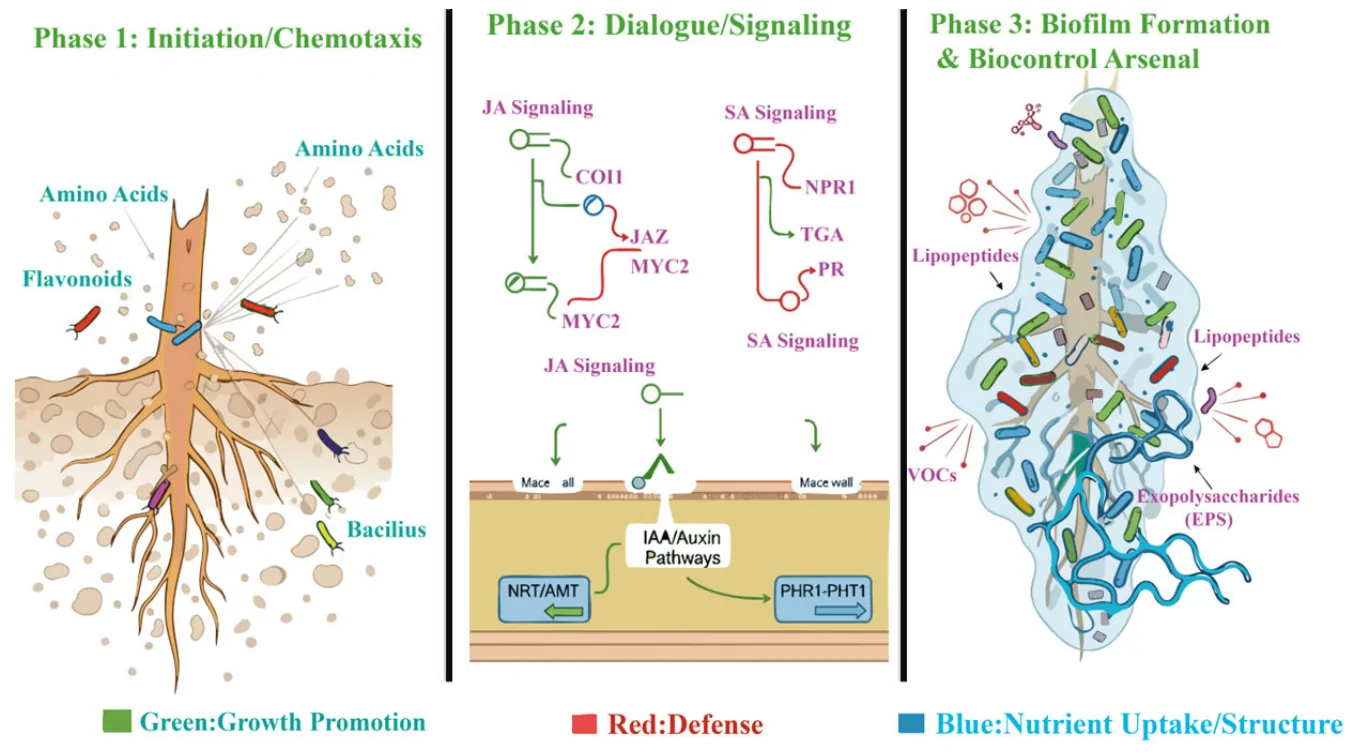
Schematic overview of the three-stage molecular interplay between PGPR and maize roots: colonization initiation, signaling crosstalk, and metabolite-mediated effects. The panel explicitly distinguishes established mechanisms (e.g., JA/SA signaling pathways, solid lines within the cells) from proposed interactions and overall field-level phenotypic outcomes.

Mechanistic dissection lies at the core of this review. The interaction between PGPR and maize roots is a temporally and spatially coordinated process (Figure 2). This process can be summarized in three key stages:

Root exudates orchestrate chemotaxis, facilitating successful rhizosphere colonization, which ultimately results in better plant growth;

Microbial signals trigger phytohormonal pathways and nutrient transport reprogramming, thereby reshaping host plant physiology;

Secondary metabolites act as multifaceted effectors that simultaneously suppress disease symptoms, activate defense responses, and ameliorate the rhizosphere microenvironment.

These processes are examined below in five areas: nutrient supply, phytohormone regulation, stress tolerance, rhizosphere colonization, and molecular signaling. Representative microorganisms are used as examples. In this section, we explicitly distinguish between direct experimental evidence, indirect associations, and proposed mechanisms. Our wording is carefully calibrated to reflect these differing levels of scientific certainty.

### 3.1. Nutrient Provisioning and Enhancement of Nutrient-Use Efficiency

Biological nitrogen fixation is catalyzed by the nitrogenase enzyme complex, which comprises the iron-protein reductase (NifH_2_) containing a [4Fe-4S] cluster and the molybdenum–iron protein (NifD_2_K_2_) harboring the P-cluster and FeMo cofactor, working in concert to fix atmospheric N_2_ to ammonia. All diazotrophs carry the conserved *nifHDK* gene cluster [24,36]; additionally, *nifU* and *nifS* participate in iron–sulfur cluster assembly. In *A. vinelandii*, overexpression of these genes coupled with enhanced electron transfer has been shown to boost nitrogenase activity by 10-fold [24,37]. In the context of phosphate solubilization, the concentration of plant-available orthophosphate in soils rarely exceeds 1 µM. Gram-negative bacteria secrete gluconic acid via the pyrroloquinoline quinone (PQQ)-dependent glucose dehydrogenase pathway to mobilize otherwise unavailable phosphorus pools; notably, *pqqC* and *pqqE* have been validated as molecular markers for predicting phosphate-solubilizing capacity and for rhizosphere tracking [33,38]. In maize/peanut intercropping systems, phosphorus fertilization combined with intercropping synergistically reshapes the rhizosphere microbiome, elevating the abundance of phosphorus-cycling genes such as *phoD* and *phoX*, and consequently boosting grain yield [39]. Furthermore, high concentrations of organic acids secreted by rhizosphere microorganisms can attain the threshold required for mobilizing unavailable phosphorus within localized microsites [40]. Potassium-solubilizing bacteria, on the other hand, disrupt the crystal lattice of potassium-bearing minerals through the secretion of organic acids and extracellular polysaccharides, thereby converting fixed potassium into plant-absorbable forms [41]. Siderophores—such as pyoverdine, which exhibit an exceptionally high affinity for Fe^3+^ (up to 10^32^ M^−1^)—serve a dual function by mobilizing iron nutrition while concurrently inhibiting the growth of phytopathogens [42,43,44].

### 3.2. Phytohormone Secretion and Ethylene Modulation

IAA constitutes the predominant phytohormone within the microbial secretome. In bacteria, approximately 90% of IAA production is channeled through the indole-3-pyruvic acid (IPyA) pathway, wherein *ipdC*—encoding indole-3-pyruvate decarboxylase—serves as the rate-limiting enzyme, whose expression is subject to cooperative regulation by the MarR-family protein MibR and the LuxR-family protein LibR [45,46]. *Enterobacter cloacae* Mz49, isolated from the maize rhizosphere, harbors a complete IAA biosynthetic gene cluster (*iaaT*, *ysnE*, and *ipdC*) and achieves an in vitro IAA yield of 64.89 μg/mL, suggesting that IAA-synthesis genes may serve as effective molecular markers for screening PGPR candidates in maize [47]. Once secreted, microbial IAA acts through the host AUX/IAA-ARF signaling cascade to promote lateral root initiation and root hair proliferation, thereby expanding the root absorptive surface area. Cytokinins—synthesized by isopentenyl transferase (IPT)—promote cell division and delay leaf senescence, whereas gibberellins stimulate stem elongation; collectively, these two hormone classes operate in concert to optimize both vegetative growth and reproductive development in maize [48,49].

Beyond auxin, cytokinin, and gibberellin signaling, ethylene modulation constitutes another critical dimension of PGPM-mediated growth promotion. Under stress conditions, elevated ethylene levels inhibit root system development and accelerate premature senescence. PGPR strains that produce ACC deaminase—encoded by *acdS*—counteract this effect by cleaving the ethylene precursor ACC into ammonia and α-ketobutyrate, thereby effectively lowering endogenous ethylene titers [50]. Transcriptomic evidence has revealed that such strains induce the upregulation of drought-tolerance genes such as *OTS1* and *ADH2* under water-deficit conditions, whereas upon rehydration, they activate recovery-associated genes including *RAD6* and *WRKY72* [50]. A systematic screening of maize-associated isolates identified ACC de-aminise activity in 38 *Bacillus* strains, among which *Bacillus velezensis* CNPSo 2384 and *Bacillus subtilis* CNPSo 2606 conferred marked improvement in drought tolerance in maize [51]. Furthermore, microbial communities harboring *acdS* have been shown to establish stable colonization in the maize rhizosphere [28,52].

### 3.3. Enhancement of Stress Tolerance

In the context of salinity stress, maize growth is constrained by osmotic imbalance, ionic toxicity, and oxidative damage. Inoculation with *Serratia liquefaciens* KM4 has been shown to upregulate ion transporter genes—including *HKT1* and *NHX1*—in both roots and leaves of maize, facilitating Na^+^ efflux and vacuolar compartmentalization, thereby maintaining a lower Na^+^/K^+^ ratio, while concomitantly downregulating *NCED* to curtail stress-induced ethylene accumulation [53]. Moreover, multi-strain consortium inoculation (comprising *Priestia megaterium*, *Pseudomonas fluorescens*, and *Bacillus subtilis*) exhibited synergistic effects superior to single-strain applications in enhancing antioxidant capacity under salt stress [54]. A 2026 study further confirmed that *Priestia megaterium* (formerly *Bacillus megaterium*) mutant SLD488, generated by atmospheric and room temperature plasma (ARTP) mutagenesis, achieved rhizosphere colonization at 2.46-fold that of the wild-type strain in saline–alkali soil, and increased maize shoot fresh weight by 40.5%, providing a novel strategy for the directed breeding of salt-tolerant growth-promoting microbial inoculants [55]. Notably, a recent study demonstrated that co-applying artificial humic acid with a multifunctional inoculant (*Bacillus* spp. and *Pseudomonas* sp.) synergistically improved saline–alkali soil conditions and increased crop yield, driven by the restructuring of bacterial communities and SOC degradation [56].

In the context of drought stress, *Pseudomonas putida* KT2440 has been shown to specifically “pre-activate” the plant’s transcriptional drought response through biofilm formation, resulting in an approximately 360% increase in the number of differentially expressed genes [57]. Additionally, PGPR and AMF act in concert to scavenge excess reactive oxygen species by enhancing antioxidant enzyme activities, thereby sustaining cellular redox homeostasis and ensuring yield formation under limited water availability [58]. With regard to biotic stress defense, rhizosphere-colonizing bacteria release signaling molecules—including lipopeptides, siderophores, and volatile organic compounds (VOCs)—that pre-trigger systemic immunity (ISR) throughout the plant [59]. Specifically, the accumulation of hydroxycinnamic acid amides and the rapid initiation of jasmonic acid signaling have been identified as pivotal events in the establishment of this systemic defense [60,61]. Moreover, combined inoculation with PGPR and AMF cooperatively activates multiple hormone signaling pathways—JA, SA, and ET—to construct a more robust systemic defense network [62].

### 3.4. Rhizosphere Colonization and Microenvironmental Modulation

Colonization constitutes a prerequisite for microorganisms to exert their growth-promoting functions. Maize roots actively recruit beneficial microbes by secreting a diverse array of metabolites, including amino acids, flavonoids, and organic acids [63,64]; the composition and efflux of these root exudates are precisely governed by transporters such as UMAMIT30 and the NSP2-MYB40 regulatory module [65,66]. Upon chemotactic migration along chemical gradients toward the root surface, initial attachment is mediated by unipolar polysaccharides (UPPs) and the XadA2 adhesin [67,68]. A small RNA, LapS—transcribed from an internal region of the *lapA* gene—post-transcriptionally modulates the expression of the LapA adhesin, thereby coordinating quorum sensing and biofilm development, which collectively determine the stability of root colonization [69]. Once established, the colonizing microorganisms can further invade root tissues through surface wounds or intercellular spaces, and exploit the vascular system for systemic dissemination throughout the plant [70].

At the level of rhizosphere microenvironmental modulation, extracellular polymeric substances (EPS) function as a “biological glue” that promotes the formation of macroaggregates (>0.25 mm in diameter), thereby improving soil aeration and water-holding capacity [71,72]. Inoculation with composite PGPR formulations has been shown to substantially increase the activities of rhizosphere sucrase, alkaline phosphatase, and dehydrogenase, along with the contents of available nitrogen and phosphorus [73,74]. Furthermore, inoculation with *Bacillus subtilis* 8-32 restructured the rhizosphere community architecture, enriching beneficial *Pseudomonas* populations while simultaneously excluding pathogenic contact with root surfaces through biofilm-mediated spatial occupation [75]. A 2025 field-based study further demonstrated that application of a bioformulation containing *Bacillus subtilis* and *Microbacterium* spp. significantly altered the bacterial community structure in the maize rhizosphere across the growing season, with a concurrent increase in microbial diversity [76].

### 3.5. Defense Signaling Pathways and Secondary Metabolite Regulatory Networks

At the level of defense signaling, the jasmonic acid (JA) pathway operates through the conjugation of JA-Ile with the COI1-JAZ coreceptor, which triggers JAZ protein degradation and releases the transcription factor MYC2. This cascade is precisely modulated by the MYC2-MED25 module, which governs both the initiation and termination of defense responses [77]. Comparative transcriptomic analyses have revealed that JA pathway genes and terpenoid metabolic genes are synergistically upregulated in gray leaf spot-resistant maize lines following inoculation with *Cercospora zeina* [78]. Moreover, non-canonical OPDA metabolites have been shown to activate marker genes such as *ZAT10* and *ERF5* independently of the COI1-JAZ receptor, thereby extending the complexity of the JA signaling network [79]. In the salicylic acid (SA) pathway, signaling relies on NPR1, which undergoes a conformational transition from an oligomeric to a monomeric state upon activation, followed by nuclear translocation and subsequent interaction with TGA transcription factors to activate the expression of *PR* genes [80,81]. In some monocot crops, OsNH2 has been identified as a regulator of SA-mediated defense signaling [82]. Furthermore, SA exerts antagonistic crosstalk with auxin signaling through the suppression of *GH3.1* [83].

At the nutrient regulatory level, microorganisms modulate nitrogen transport through the NRT/AMT family, where external pH exerts interactive co-regulation over nitrate versus ammonium uptake [84]; moreover, microbial volatile organic compounds (VOCs) have been shown to upregulate the expression of *NRT1.2* and *NRT2.1* [85]. In parallel, the PHR1-PHT1 phosphorus acquisition module is subject to microbial modulation, with MmPHR1 directly binding to the promoter of *MmPHT1;5* to activate its expression [86], and the UrMYB62-*UrPHT1;11* regulatory axis functioning as a key responsive cascade under low-phosphate stress [87].

At the level of secondary metabolites, *Bacillus* spp. synthesize three major classes of lipopeptides—surfactin, iturin, and fengycin. This occurs via the non-ribosomal peptide synthetase (NRPS) pathway, wherein the amino acid specificity of the adenylation domains dictates the composition of the peptide chain, enabling targeted engineering through domain swapping [88,89]. Volatile organic compounds (VOCs), including acetoin and 2,3-butanediol, not only directly promote nutrient uptake and salt tolerance but they also reprogram the rhizosphere microbial community by enriching nitrogen-fixing and phosphate-solubilizing functional guilds, and they can trigger plant “immune memory” [90,91,92]. Whole-genome sequencing has revealed that strains such as *Bacillus velezensis* harbor multiple biocontrol gene clusters alongside functional genes for chemotaxis, biofilm formation, nitrogen fixation, and phosphate solubilization, providing a suite of genetic markers for the targeted screening of maize-specific inoculant strains [93,94]. The key molecular mechanisms governing maize–microbiome interactions are summarized in Table 2.

All strain traits, numerical values, and reported phenotypes in Table 1, Table 2 and Table 3 are directly extracted from the corresponding cited sources, and any inferred regulatory relationships are explicitly described in the main text.

## 4. Multi-Omics Perspectives on the Maize–Microbiome Interaction Network

The integrative application of multi-omics technologies has emerged as a cornerstone for dissecting the maize–microbiome interplay, and constitutes the distinguishing feature that sets this review apart from earlier syntheses. In the following sections, we systematically survey this rapidly advancing frontier across three complementary levels—genomics, transcriptomics/epigenomics, and metagenomics/metabolomics—with particular emphasis on the G×M×E interaction network, which we identify as the central bottleneck constraining field-scale translation. However, integrating these datasets is constrained by batch effects and limited statistical power in heterogeneous field trials. While genomics, transcriptomics, and metabolomics respectively reveal genetic potential, active expression, and biochemical phenotypes, the resulting co-occurrence networks often indicate correlation rather than causality. Therefore, rigorous functional validation (e.g., gene knockout and multi-season field trials) remains indispensable to establish reproducible causal mechanisms.

### 4.1. Genomics and Comparative Genomics

At the genomic level, comparative genomic analyses of *Pseudomonas fluorescens* populations have revealed that gene clusters involved in siderophore synthesis, phytohormonal regulation, phosphate solubilization, and antimicrobial compound production are widely distributed across both disease-suppressive and disease-conductive soils; however, functional redundancy and environmentally dependent expression ultimately determine the actual growth-promoting contribution of individual strains [104]. The high-quality genome of the salt-tolerant endophytic fungus *Tetraploa* sp. E00680 revealed an expanded tryptophan metabolic pathway, which may enable cross-kingdom metabolic coupling with host auxin biosynthesis, constituting a molecular basis for salt tolerance and yield enhancement in plants [105]. A 2026 whole-genome analysis of *Priestia* sp. RMT2NF4 uncovered a complete set of plant growth-promoting functional gene clusters, including genes for IAA synthesis, phosphate solubilization, siderophore production, and ACC deaminase, providing a genomic foundation for strain functional prediction [19]. Importantly, genomic prediction of a functional trait does not establish that the pathway is expressed, that the strain colonizes the rhizosphere successfully, or that the trait contributes to yield under field conditions. Therefore, conclusions drawn from genome mining alone should be interpreted as hypotheses requiring biochemical, colonization, and field-level validation.

### 4.2. Transcriptomics and Epigenomics

At the transcriptomic and epigenomic levels, weighted gene co-expression network analysis (WGCNA) has identified co-expression modules positively correlated with resistance to gray leaf spot in maize, wherein terpenoid metabolism and jasmonic acid (JA) signaling genes cooperatively reinforce defense responses [78]. In the early stages of combined inoculation, DNA hypomethylation has been pinpointed as a key epigenetic hallmark [34]. The advent of single-cell RNA sequencing and spatial transcriptomics has further made it feasible to dissect, at cellular resolution, the processes of microbial perception, immune activation, and symbiotic differentiation programs in plants [106].

### 4.3. Integrative Metagenomics and Metabolomics

Integrated metagenomic and metabolomic analyses have confirmed that *Bacillus*-produced VOCs function as signaling molecules that recruit rhizobacterial taxa with nitrogen-fixing, phosphate-solubilizing, and growth-promoting capabilities, while simultaneously reshaping the metabolite profiles of both host roots and leaves—revealing a bidirectional plant–microbe dialogue mediated by volatile compounds [90]. A 2025 field study demonstrated that inoculation with a bioformulation significantly altered the bacterial community composition in the maize rhizosphere; using 16S rRNA sequencing, the researchers observed an increased abundance of beneficial genera [76]. A 2026 study further showed that a staged microbial consortium effectively converted cattle manure wastewater into a bioactive fermentation broth, with metabolomic profiling uncovering a “pre-modification followed by synergy” mechanism underlying its growth-promoting effects [107].

### 4.4. G×M×E Interaction Network and Translational Bottlenecks in Field Application

At the network level, multi-omics data enable the integration of microbial strains, metabolic pathways, and maize physiological responses into a cohesive interaction network: host genotype selectively recruits specific microbial taxa such as Bacillaceae through root metabolites (e.g., kaempferol), and these microorganisms in turn promote root development and nutrient uptake via the jasmonic acid/ethylene signaling pathway, thereby elucidating the molecular mechanisms by which plant genetic background governs rhizosphere microbiome assembly [108]. Specifically, host genotype (G) determines the efficacy of inoculation methods, as multiple applications are often required to overcome genotype-specific barriers under low-nitrogen conditions [109]. Furthermore, native soil microbiome (M) and environmental fluctuations (E) dictate the colonization success of inoculants, often overriding the intrinsic potential observed in laboratory monocultures [75,109,110]. Predictive models based on pre-planting soil microbiome diagnostics have been shown to account for 38% of the variation in yield response to microbial inoculants [111]. Overall, these results suggest that G×M×E interactions are a major reason why laboratory findings often do not translate into stable field results, highlighting the critical need for comprehensive field trials. Future efforts should focus on systematically dissecting G×M×E interaction patterns through integrated multi-omics and machine learning modeling, thereby providing decision support for site-specific inoculant deployment.

To provide a clearer overview of the multi-omics toolbox and its practical implementation, we have summarized the primary methodologies, data types, limitations, and required validation approaches in Table 3.

## 5. Synthetic Microbial Consortia and Field Application

### 5.1. Synergistic Mechanisms and Application Advantages of Synthetic Consortia

In current maize cultivation, virtually all field-scale applications of beneficial microorganisms rely on multi-strain formulations rather than single isolates—a consensus that has become widely acknowledged within the industry. Synthetic microbial consortia, composed of two or more microbial species with complementary functionalities (including cross-kingdom combinations of PGPR and PGPF), offer distinct advantages over single-strain inoculants, including functional diversification, enhanced environmental adaptability, and more consistent field performance [112]. Among the various combinations, co-inoculation of AMF with bacteria represents the most extensively investigated paradigm [26,27]; similarly, combined application of *Trichoderma harzianum* and *Bacillus subtilis* has been shown to induce systemic resistance in plants via DNA hypomethylation [34,35]. A 2026 study on a staged microbial consortium that converts cattle manure wastewater into a growth-promoting fermentation broth further validated the efficacy of the “pre-modification followed by synergy” strategy in synthetic consortia design [107]. It should be cautioned, however, that the synergistic effects observed between co-inoculated strains are highly environmentally contingent; positive outcomes documented under controlled conditions frequently fail to translate into open-field settings, owing to competitive exclusion and niche overlap. Moreover, practical deployment is constrained by inter-strain antagonism, colonization resistance from native microbiomes, formulation and shelf-life instability, and higher production costs relative to single-strain inoculants. However, the optimal strain combinations for specific maize genotypes and distinct agro-ecological zones remain largely undefined, underscoring the pressing need for genotype-by-microbiome matching strategies [35].

### 5.2. Application Methods and Agronomic Performance

The application methods of microbial fertilizers in maize production have become increasingly diversified (Table 4). Seed coating facilitates early root colonization and improves rhizosphere community establishment; however, its growth-promoting efficacy is significantly influenced by maize genotype, and under low-nitrogen conditions, multiple inoculations outperform single application [113]. Rhizosphere irrigation, which delivers bacterial suspensions directly to the root zone, substantially increases colonization density and exhibits pronounced synergistic effects when combined with organic fertilizers [76]. Soil application of synthetic microbial consortia (comprising nitrogen-fixing bacteria, phosphate-solubilizing bacteria, and beneficial communities) has been shown to improve crop productivity under recommended fertilizer rates, while concurrently improving soil enzyme activities and community structure [114]. Bio-organic fertilizers enhance soil organic carbon content, ameliorate physicochemical properties, and increase silage maize yield [115,116]. Co-application of AMF and biochar significantly improves maize growth under salt stress [27]. Furthermore, integrated biofertilizer and organic amendment approaches provide a paradigm for multi-level evidence linking biochemical changes to agronomic benefits. For instance, Rafiq et al. [117] demonstrated that combining commercial biofertilizers with organic amendments significantly reduced black scurf disease and increased tuber weight in potatoes, which was directly supported by increased activities of defense-related enzymes (CAT, POX, and PPO). This highlights the necessity of phenotypic and biochemical validation beyond mere genomic prediction. A 2023 study confirmed that combined biochar and biofertilizer application reduces the requirement for nitrogen fertilizer while increasing yields in maize–soybean relay strip intercropping systems [118]; a 2026 investigation of biochar–earthworm–plant interactions further revealed synergistic mechanisms underlying soil organic carbon stabilization and cadmium immobilization in maize cropping systems [119]. Compared with conventional fertilizer management, synthetic consortia can maintain crop yields with reduced chemical inputs, though their persistence is highly dependent on application dose and timing [113]. In contrast, single-strain inoculants often perform well in greenhouses but fail in replicated field trials due to native microbial competition. While they offer better shelf-life, they lack the functional redundancy of multi-strain consortia [76,113].

### 5.3. Industry Status and Standardization Challenges

As of 2026, the commercialization of microbial fertilizers continues to expand, though regulatory frameworks vary significantly across regions. In China, thousands of microbial fertilizer products have been registered under a centralized regulatory system administered by the Ministry of Agriculture and Rural Affairs (https://flyw.agri.cn/publicvue/#/Ministerial_query) [115]. In contrast, a 2024 study highlighted that the United States lacks a unified federal framework, with oversight fragmented across the EPA, USDA-APHIS, and state-level fertilizer laws [109]. These divergent governance models highlight the lack of a global standardized evaluation system. Despite this expansion, the industry still faces persistent challenges, including inconsistent product performance and inadequate field-evaluation standards [76].

## 6. Persistent Challenges and Future Perspectives

### 6.1. Persistent Challenges

First, single-strain inoculants often suffer from narrow functionality and poor field stability. The pronounced physicochemical heterogeneity of the soil environment severely constrains the active dispersal of bacteria [110]; temperature, pH, and incubation time significantly affect strain viability and stress tolerance, with the physiological status of the inoculant being a critical determinant of colonization success [121]. Moreover, the plant defense responses elicited by different strains are highly strain-specific, and growth-promoting outcomes are co-determined by the strain, the host genotype, and the stress type, which further exacerbates the unpredictability of field performance [122].

Second, the current research landscape remains fragmented. A substantial gap persists between controlled experiments and field outcomes. Traditional culture-based approaches may overlook rare but functionally important taxa [123]; moreover, the assembly rules and functional compensation mechanisms of the rhizosphere microbiome under salt stress and other constraints remain poorly understood, and a predictive framework capable of translating controlled-environment findings into field applications is still lacking [124,125].

Third, the lack of industrial standardization poses a major hurdle. Traditional peat-based carriers are unsustainable, while liquid formulations incur high storage and transportation costs and exhibit poor environmental tolerance [126]. The development of protective agents for complex formulations and the maintenance of adequate shelf-life remain challenging [127,128].

Fourth, the biosafety and environmental risks of strain release require rigorous assessment. Plant growth-promoting potential alone does not establish that an organism is suitable for agricultural release. Notably, several widely studied PGPR taxa possess dual-use traits; for instance, although *Bacillus* and *Pseudomonas* are core beneficial taxa [15], certain strains within the *Bacillus cereus* group [5] and the genus *Ochrobactrum* [11] can be opportunistic pathogens. Additionally, introducing exogenous strains at high densities can disrupt native soil microbiomes [75,76]. Consequently, rigorous strain-level risk assessments—including genomic screening for virulence determinants and evaluating non-target ecological impacts—are prerequisites for safe agricultural deployment [129,130].

### 6.2. Future Research Directions

Future endeavors should synergistically advance across three interconnected pillars: synthetic microbial consortia development, multi-omics-driven mechanistic dissection, and site-specific deployment decision-making. To translate mechanistic insights into practical field applications, future research should prioritize four actionable areas:

(1) Strain selection and optimization: Transitioning from conventional phenotype-based screening to genomics-driven high-throughput prediction to mine elite strains, coupled with adaptive laboratory evolution to enhance metabolic robustness.

(2) Multi-location field trials: Conducting rigorous, multi-location and multi-season field validations to evaluate strain persistence and efficacy across diverse agro-ecological zones.

(3) Formulation and delivery: Developing sustainable carriers (e.g., agricultural wastes, compost, biochar) and high-performance liquid formulations with extended shelf-life to replace conventional peat-based products.

(4) Predictive deployment: Establishing microbiome-diagnostic decision pathways and machine learning models to enable site-specific inoculant deployment, providing a reliable guide for field extrapolation [131,132,133].

On the mechanistic front, the integration of genomic, transcriptomic, metabolomic, and single-cell/spatial omics data should be employed to systematically reconstruct a multi-layered interaction network bridging microbial strains, metabolic pathways, and maize physiological responses, thereby elucidating the G×M×E interaction patterns and providing a theoretical foundation for translating mechanistic understanding into consistent field performance [90,105,106]. Furthermore, the establishment of a comprehensive policy framework covering product quality standards, regulatory systems, and farmer training and extension programs will serve as the institutional guarantee for the large-scale deployment of microbial fertilizers [129,130].

## 7. Conclusions

This review presents a systematic synthesis of the functional guilds, molecular mechanisms, multi-omics networks, and field application status of growth-promoting microorganisms in the maize rhizosphere. *Bacillus*, *Pseudomonas*, diazotrophs, arbuscular mycorrhizal fungi, and *Trichoderma* constitute the core functional microbiome associated with maize yield enhancement, with synthetic microbial consortia increasingly being adopted as a predominant approach in field application owing to strain–strain synergies. Microorganisms may promote maize yield through four principal pathways: (1) optimizing nutrient provisioning via biological nitrogen fixation, phosphate solubilization, potassium mobilization, and iron activation; (2) regulating plant growth and development through secretion of IAA, cytokinins, gibberellins, and ACC deaminase; (3) enhancing stress tolerance by modulating ion homeostasis, antioxidant defense, and induced systemic resistance; (4) improving the rhizosphere microenvironment through exopolysaccharide production and community modulation. However, it is important to note that improvements in early-stage traits—such as germination rates, root biomass, and nutrient uptake—along with disease suppression, are intermediate phenotypes. While these mechanisms underpin yield formation, they do not always translate directly into consistent grain yield increases under field conditions. The molecular underpinnings involve complex cross-talk between microbial functional genes—including *nif*, *pqq*, and *acdS*—and plant signaling modules such as the JA/SA defense pathways, NRT/AMT nitrogen transporters, and the PHR1-PHT1 phosphorus acquisition system. Multi-omics and network analyses provide a systematic framework for dissecting G×M×E interactions and overcoming translational bottlenecks. Recent advances from 2023 to 2026 have made significant strides in directed breeding of salt-tolerant strains [55], single-spore propagation of native AMF [28], genomic dissection of plant growth-promoting *Priestia* strains [19], staged synthetic consortia design [107], biochar–biofertilizer synergistic nitrogen reduction [118], and regulatory frameworks for biofertilizers [109]. Further advances in synthetic microbial consortia, multi-omics-based mechanistic studies, and site-specific deployment, together with improved standards and policy support, are expected to facilitate the wider application of microbial fertilizers in sustainable maize production. Although further multi-location field validations remain necessary, these advances hold promise for reducing reliance on synthetic fertilizers and enhancing the resilience of maize cropping systems.

## Figures and Tables

**Table 1:** Core microbial taxa associated with maize yield enhancement and their suggested functional mechanisms.

Microbial Taxa	Key Functions	Representative Strains	Suggested Mechanism	Ref.
*Bacillus* spp.	Biocontrol, PGP, nutrient solubilization	*B. velezensis* HN-Q-8, *B. subtilis*, *B. amyloliquefaciens*	Lipopeptide antibiotics (fengycin, iturin, surfactin), IAA, siderophores, phosphate solubilization	[15,16,17,18,31,32]
*Pseudomonas* spp.	Iron acquisition, pathogen suppression, PGP	*P. aeruginosa*, *P. fluorescens* MFE01, *P. putida* RB-2	Pyoverdine/pyochelin siderophores, GacS/GacA-regulated T6SS, VOCs (1-undecene, HCN), *pqqE* marker for P-solubilizing Gram-negatives	[20,21,22,33]
Nitrogen-fixing bacteria	BNF, phytohormone production, P uptake	*A. brasilense* Sp245, *A. vinelandii*	NifHDK nitrogenase complex, IAA via IPyA pathway, upregulation of *PHT1;1*, *PHT1;4*, *PHO1*	[23,24]
Arbuscular mycorrhizal fungi (AMF)	Nutrient uptake enhancement, stress tolerance	*R. irregularis*	Hyphal network expansion, P/N uptake via symbiotic interface, synergistic P nutrition with PSB	[25,26,27,34]
*Trichoderma* spp.	Biocontrol, growth promotion	*T. harzianum* K179	Antifungal activity against *F. graminearum* and *A. flavus*, ROS burst via JA signaling, mycotoxin reduction	[29,30]
Composite inoculants	Synergistic growth promotion, enhanced adaptability	AMF + *B. subtilis*, *T. harzianum* + *B. subtilis*	Functional diversification, microbial community restructuring, ISR priming via DNA hypomethylation	[26,35]

**Table 2:** Suggested molecular mechanisms underlying microbe–maize interactions.

Interaction Phase	Key Molecules/Processes	Regulatory Networks	Functional Outcomes	Ref.
Root colonization (Phase 1)	Root exudates (amino acids, flavonoids, organic acids), chemotaxis, UPP-mediated attachment, LapA biofilm regulation	UMAMIT30 (amino acid export), NSP2-MYB40 (flavonoid biosynthesis), quorum sensing	Recruitment and stable colonization of beneficial microbes	[63,64,65,66,67,68,69,95]
JA signaling	JA-Ile, COI1-JAZ co-receptor, MYC2-MED25 module, non-canonical OPDA metabolites	*ZAT10*, *ERF5*, terpenoid metabolism; CsCOI1–PIF7 growth–defense trade-off	Defense against necrotrophic pathogens (e.g., *C. zeina*), gray leaf spot resistance	[77,78,79,96,97]
SA signaling	NPR1 oligomer–monomer transition, TGA transcription factors, MED19a mediator	*PR1*, *PR3*, *PR5*, *OsNH2*; NPR1-NPR3 functional divergence	Systemic acquired resistance (SAR), antagonistic crosstalk with auxin via *GH3.1*	[80,81,82,83,98]
Nitrogen uptake	NRT1.1/NRT2.1 (nitrate), AMT (ammonium), microbial VOCs	Reciprocal co-regulation by external pH, VOC-mediated *NRT1.2* upregulation	Enhanced N acquisition, lateral root formation under N deficiency	[84,85,99]
Phosphorus uptake	PHR1-PHT1 module, *MmPHR1*–*MmPHT1;5*, acid phosphatase	Direct promoter binding, MYB62–*UrPHT1;11* regulatory axis	Improved P mobilization and uptake under low-P conditions	[86,87]
Secondary metabolites	Lipopeptides (NRPS), VOCs (acetoin, 2,3-butanediol), EPS	NRPS/PKS gene clusters, phenylpropanoid pathway activation, biofilm matrix stabilization	Multi-dimensional pathogen suppression, rhizosphere microbiome reprogramming, soil aggregate formation	[88,89,90,91,92,93,94,100,101,102,103]

**Table 3:** Comparison of multi-omics approaches for dissecting maize–microbiome interactions.

Omics Approach	Data Types Generated	Key Scientific Questions Addressed	Major Limitations	Required Validation
Genomics	Whole-genome sequences, biosynthetic gene clusters (BGCs)	What is the genetic potential of a strain or community?	Does not prove gene expression; silent or cryptic gene clusters; functional redundancy.	Gene knockout/mutation, heterologous expression, in vitro biochemical assays.
Transcriptomics & Epigenomics	mRNA transcripts, non-coding RNAs, DNA methylation patterns	Which genes are actively expressed and how are they regulated?	High sensitivity to environmental fluctuations; RNA degradation; poor correlation with protein abundance.	qRT-PCR, proteomics, targeted metabolite analysis, in planta expression validation.
Metagenomics	Total DNA from environmental samples	Who is there, and what functional genes are enriched?	DNA from dead cells; biases in DNA extraction; limited resolution for strain-level tracking.	Metatranscriptomics, stable isotope probing (SIP), qPCR, culturomics.
Metabolomics	Primary and secondary metabolites (VOCs, phytohormones)	What biochemical phenotypes are produced during the interaction?	Rapid turnover of metabolites; matrix effects in complex soil; difficult to attribute to specific microbes.	Isotopic labeling, targeted metabolite quantification, in vitro bioassays.
Single-cell/Spatial Omics	Cell-specific transcriptomes, spatial localization maps	How do individual plant cells respond to microbial cues?	High cost; challenging sample preparation; lack of robust reference genomes for complex microbiomes.	In situ hybridization (FISH), reporter gene assays, spatial proteomics.
Multi-omics Integration	Integrated correlation networks, machine learning models	How do genes, transcripts, and metabolites interact as a system?	Batch effects; limited statistical power; co-occurrence networks only indicate correlation, not causality.	Multi-season field trials; synthetic community reconstitution; functional validation (e.g., gene knockout).

**Table 4:** Application methods and agronomic performance of microbial biofertilizers in maize production.

Application Method	Target Microorganisms	Key Effects on Maize	Yield Response	Ref.
Seed dressing	*Bacillus* spp., *Pseudomonas* spp.	Early root colonization, improved rhizosphere community establishment, genotype-dependent growth promotion	Variable; multiple inoculations outperform single application under low-N conditions	[113]
Root irrigation	*B. subtilis*, *Microbacterium* spp.	Enhanced rhizosphere colonization density, improved community structure and diversity, synergistic with organic fertilizer	Yield increase 8–15%; N fertilizer reduction 20–30%	[76,120]
Soil application (composite inoculants)	N-fixers + P-solubilizers + beneficial communities	Improved soil enzyme activity, enhanced microbial community structure, reduced chemical fertilizer dependence	Yield increase 8–15% with recommended chemical fertilizer doses	[114]
Bio-organic fertilizer	Mixed PGPR consortia + organic amendments	Enhanced soil organic C (+0.44 g C kg^−1^ soil), improved physicochemical properties, increased silage maize yield	Significant yield enhancement under field conditions	[115,116]
AMF + biochar co-application	*R. irregularis* + biochar	Alleviated salt stress, increased K, Ca, and Mg uptake	Significant growth improvement under salinity stress	[27]

## Data Availability

No new data were created or analyzed in this study.
